# Supplementary material for: Greenhouse warming and internal variability increase extreme and central Pacific El Niño frequency since 1980
Source: Nat Commun. 2023 Jan 24;14:394. doi: 10.1038/s41467-023-36053-7 (PMC9873625; doi:10.1038/s41467-023-36053-7)
Supplement: Supplementary file 1 — Supplementary Information [file 41467_2023_36053_MOESM1_ESM.pdf]

Supporting Information for

Greenhouse warming and internal variability increase extreme and central Pacific El Niño frequency since 1980

Ruyu Gan[1,2,3], Qi Liu[4,5*], Gang Huang[1,2,3*], Kaiming Hu[1,6], and Xichen Li[7]

[1]*State Key Laboratory of Numerical Modeling for Atmospheric Sciences and Geophysical Fluid Dynamics and Center for Monsoon System Research, Institute of Atmospheric Physics, Chinese Academy of Sciences, Beijing, China*

[2]*Laboratory for Regional Oceanography and Numerical Modeling, Qingdao National Laboratory for Marine Science and Technology, Qingdao 266237, China*

[3]*University of Chinese Academy of Sciences, Beijing 100049, China*

[4]*School of Atmospheric Sciences, Nanjing University, Nanjing 210023, China.*

[5]*Joint International Research Laboratory of Atmospheric and Earth System Sciences, Nanjing University, Nanjing 210023, China.*

[6]*Collaborative Innovation Center on Forecast and Evaluation of Meteorological Disasters (CIC-FEMD), Nanjing University of Information Science & Technology, Nanjing, China*

[7]*International Center for Climate and Environment Sciences, Institute of Atmospheric Physics, Chinese Academy of Sciences, Beijing, China*

***Corresponding authors: Qi Liu and Gang Huang**

(Qi Liu) Joint International Research Laboratory of Atmospheric and Earth System Sciences, School of Atmospheric Sciences, Nanjing University, Nanjing 210023, China. E-mail: qiliu@nju.edu.cn

(Gang Huang) State Key Laboratory of Numerical Modeling for Atmospheric Sciences, Institute of Atmospheric Physics, Chinese Academy of Sciences, Beijing 100029, China. Telephone: +86-10-82995312. E-mail: hg@mail.iap.ac.cn

Contents of this file:

Supplementary Discussion
Supplementary Tables 1 to 3
Supplementary Figures 1 to 11
Supplementary References

Supplementary Discussion

Sensitivity. We analyzed the sea surface temperature (SST) taken from the HadISST[1], ERSSTV5[2] and Kaplan extended version 2[3] for the period 1871-2017. Here, we used the 38 identified El Niño events using the merged HadISST, ERSSTV5, and Kaplan SST datasets. Supplementary Figure 2a-c displays the occurrence of different types of El Niño events using these three SST datasets. Similar to the results based on the merged data, frequent extreme and Central Pacific (CP) El Niño events are observed in two periods: 1875-1905 and 1980-2017. For example, for the HadISST data, 3 extreme (1877, 1888, 1902) and 3 CP (1884, 1885, 1896) El Niño events were identified in the period of 1875-1905, and 3 extreme (1982, 1997, 2015) and 7 CP (1986, 1994, 2002, 2004, 2006, 2009, 2014) El Niño events were identified for the period of 1980-2017. The results show that there is general agreement that extreme and CP El Niño events are common in both the periods of 1875-1905 and 1980-2017.

To test whether the classification results are sensitive to the classification method, we also used the traditional Niño method[4-5] to categorize 38 El Niño events into CP and Eastern Pacific (EP) El Niño events. An El Niño event is classified as a CP (EP) type when the DJF-averaged value of the normalized Niño4 index is greater (less) than the average value of the normalized Niño3 index. The Niño4 index equals the detrended SST anomaly averaged over 160°E–150°W, 5°S–5°N; the Niño3 index equals the detrended SST anomaly averaged over 150°W–90°W, 5°S–5°N. However, this method cannot identify extreme El Niño events directly. Therefore, we further divide the EP type El Niño events into moderate EP and extreme El Niño events by the criterion of the DJF-averaged value of the normalized Niño3 index below or above a threshold value of 1.75 standard deviations. We found that similar to the results based on the cluster analysis, there is another period (from the 1870s to 1900s) with increased CP and extreme El Niño events, like the recent decades. The difference likely arises from that our cluster method not only considers the SST spatial pattern in the El Nino peak phase but also considers SST evolution in El Nino development[6]. In addition, the frequencies of different El Niño types based on the traditional classification method exhibit significant correlations with the internal multidecadal variability (Supplementary Fig. 10e-h), and these correlations are consistent with those based on the cluster classification method of this study (Supplementary Fig. 10). Moreover, we estimate that internal variability contributed to ~77% of the increasingly extreme and CP El Niño events, while anthropogenic forcing has made our globe experience ~1.1 more extreme and ~1.2 more CP events over the past four decades (Supplementary Fig. 11). Therefore, the conclusion still holds that the recent changes in El Niño events were attributed to synchronized effects of greenhouse warming and internal variability.

We also used another classification method to test the sensitivity. We defined extreme El Niño events using EOF analysis over the equatorial Pacific (15°S–15°N and 140°E−80°W) based on monthly SST data, and CP and EP El Niño events are represented by C-index = ($PC1_{ENSO}$+ $PC2_{ENSO}$)/2 and E-index = ($PC1_{ENSO}$-$PC2_{ENSO}$)/2,

respectively[7-10]. The extreme El Niño event is identified as a DJF season-averaged E-index > 1.75 standard deviations (s.d.). Since the classification method we used focused on the temporal evolution of ENSO events, we also tested another classification method by examining the spatial–temporal evolution of El Niño based on the pioneering work of ref.[6]. A total of 27 moderate El Niño events (including EP and CP) are classified into four types, EP, Eastern Central Pacific (ECP), and Western Central Pacific (WCP) El Niño events, following the pioneering work of ref.[6] The EP is defined as the noticeable (greater than 0.5°C) initial SSTA that first appears in the Niño1+2 region and propagates westward to the International Dateline. A total of 12 EP El Niño (1876, 1899, 1904, 1911, 1913, 1918, 1923, 1925, 1951, 1957, 1965, 1976) events are identified. The ECP is defined as the noticeable initial SSTA that first appears in the eastern central Pacific (110°W–150°W) and extends both eastward and westward, and a total of 4 ECP El Niño (1896, 1930, 1963, 1968) events are identified. The WCP El Niño is defined as the noticeable initial SSTA that first appears in the western central Pacific (west of 150°W) and then propagates eastward; a total of 11 WCP El Niño events (1884, 1885, 1977, 1986, 1991, 1994, 2002, 2004, 2006, 2009, 2014) are identified. Years in bold highlight events that are identified as the same type as using the above cluster analysis. The majority of CP, EP, and extreme years coincide with years previously identified despite different classification methods. Similar to the results based on the cluster analysis, almost all moderate CP and extreme El Niño events occurred from 1875 to 1905 and 1980 to 2017. Supplementary Fig. 2d shows the classification of El Niño events, including extreme, EP, WCP, and ECP events.

Supplementary Table 1 Comparison between the period of 1875-1905 and 1981-2017 in the ocean mixed-layer heat budget analysis of 2 types of El Niño during their respective onset phases (onset phases is defined as the month when the value of the Niño-3.4 index first exceeds 0.5°C and the two months after that) over the central-eastern Pacific (5°S-5°N, 180°-80°W). The units in the ordinates are: °C month^{-1}. The most important dynamic feedback processes are the zonal advective feedback ($-u'\partial\overline{T}/\partial x$), thermocline feedback ($-\overline{w}\partial T'/\partial z$), and upwelling feedback ($-w'\partial\overline{T}/\partial z$), which are associated with the anomalous zonal currents, vertical displacement of the thermocline, and upwelling, respectively.

ENSO Types	year	$\frac{-u'\partial\overline{T}}{\partial x}$	$\frac{-\overline{u}\partial T'}{\partial x}$	$\frac{-u'\partial T'}{\partial x}$	$\frac{-w'\partial\overline{T}}{\partial z}$	$\frac{-\overline{w}\partial T'}{\partial z}$	$\frac{-w'\partial T'}{\partial z}$
extreme (1875-1905)	1877	0.20	-0.02	0.02	0.00	0.18	-0.04
	1888	0.08	-0.04	0.01	0.00	0.00	-0.03
	1902	0.44	0.01	-0.15	0.17	0.29	-0.13
CP (1875-1905)	1884	0.07	-0.01	0.00	0.06	0.05	-0.07
	1885	0.17	0.00	0.01	0.14	0.06	-0.06
	1896	0.08	-0.06	0.03	-0.05	0.06	-0.02
extreme (1980-2017)	1982	0.24	-0.02	0	0.19	0.05	-0.07
	1997	0.4	-0.07	-0.13	0.29	0.14	-0.09
	2015	0.09	0	-0.02	0.16	-0.07	-0.03
CP (1980-2017)	1986	0.29	-0.04	0	0.07	0.07	-0.02
	1991	0.08	-0.01	0	0.04	-0.03	-0.07
	1994	0.23	-0.02	0.07	0.04	-0.1	-0.05
	2002	-0.07	0.03	-0.03	-0.06	-0.11	-0.07
	2004	0.04	-0.05	0.02	-0.05	-0.03	-0.01
	2006	0.12	-0.03	0	0	0.05	-0.03
	2009	0.54	-0.04	-0.07	0.3	0.05	-0.07
	2014	0.08	-0.06	-0.1	0.04	0.02	-0.1

Supplementary Table 2 Contingent (2-way) table showing the regime change of El Niño among pre-1905, 1906-1980, and post-1980. The numbers of the 3 types of El Niño events are shown in the Tables. The degree of freedom equals (3-1)×(3-1) = 4, and the chi square value equals 16.93 ($p<0.01$).

	1875-1905	1906-1980	1981-2017	Total
extreme	3	1	3	7
CP	3	2	8	13
EP	2	10	0	12
Total	8	13	11	32

Supplementary Table 3 The number of ensemble simulations for the 27 CMIP6 models used in this study. ALL for historical all-forcing runs, GHG for greenhouse gas-only runs, NAT = natural forcing, AA for anthropogenic aerosols only runs.

No.	Model name	ALL	GHG	NAT	AA
1	BCC-CSM2-MR	3	3	3	3
2	BCC-ESM1	3			
3	CanESM5	50	50	30	30
4	CESM2	11	3	3	1
5	CESM2-WACCM	3			
6	CNRM-CM6-1	29	10	10	10
7	CNRM-ESM2-1	9			
8	E3SM-1-0	5			
9	EC-Earth3-Veg	4			
10	FGOALS-f3-L	3			
11	FGOALS-g3	3			
12	GFDL-ESM4	3	1	3	1
13	GISS-E2-1-G	30	5	5	5
14	GISS-E2-1-H	23			
15	HadGEM3-GC31-LL	4	4	4	4
16	INM-CM5-0	10			
17	IPSL-CM6A-LR	32	10	10	10
18	MIROC-ES2L	3			
19	MIROC6	10	3	3	3
20	MPI-ESM-1-2-HAM	2			
21	MPI-ESM1-2-HR	10			
22	MPI-ESM1-2-LR	10			
23	MRI-ESM2-0	6	3	3	3
24	NESM3	5			
25	NorCPM1	30			
26	NorESM2-LM	3	3	3	3
27	UKESM1-0-LL	17			
	Total number	321	95	77	73

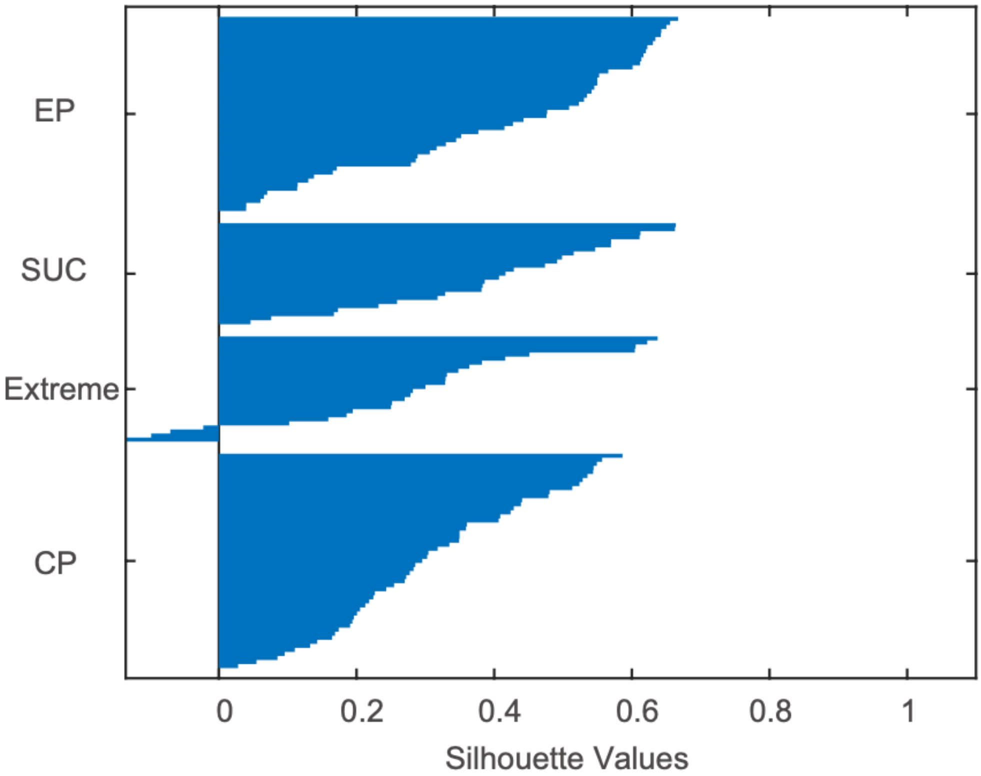

Supplementary Fig. 1 Silhouette values for each El Niño event within each of the 4 clusters for the 1871-2017 period. The silhouette value, ranging from -1.0 to +1.0, is a measure of how similar a member is to other members in its own cluster when compared to the members in other clusters. The merged SST, HadISST, ERSST5 and Kaplan datasets from 1871 to 2017 were used. Note that for the four datasets, the clusters are well separated except for 1888.

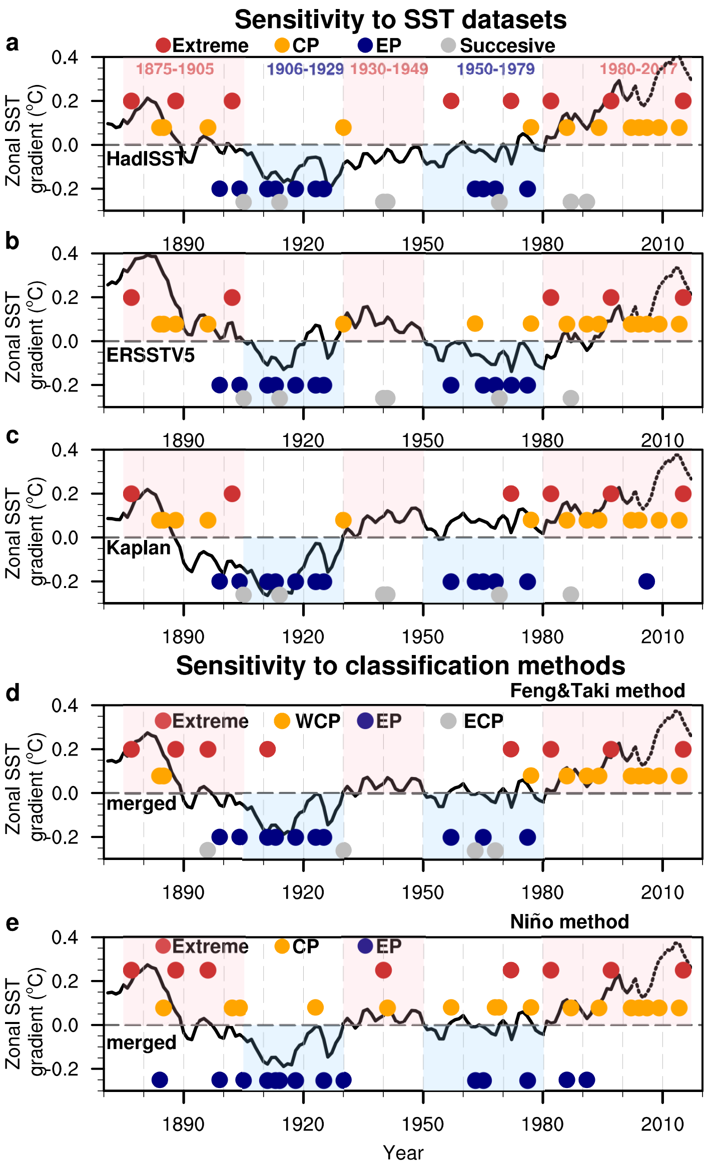

Supplementary Fig. 2 Occurrence of different types of El Niño events. a-c Sensitive to the SST datasets. (**a**) The 38 El Niño events are classified into extreme (red dots), Central Pacific (CP, orange dots), Eastern Pacific (EP, blue dots), and Successive (gray dots) events. The time series of the 31-year running mean, annual-mean zonal sea surface temperature (SST) gradient [western Pacific SST (135–165°E) minus central Pacific SST (165–145°W)] for the observations from 1871–2017 (°C, relative to the mean of 1901-2010, black line). The positive (black line is greater than the 1901-2010 mean) and negative (black line is less than the 1901-2010 mean) phases of the zonal equatorial SST gradient are represented by light red and light blue shading, respectively. The dashed lines represent not full 31-year running. **b-c** Same as **(a)** but for **(b)** ERSSTv5 and **(c)** Kaplan SST. **d-e** Sensitive to classification method. **(d)** The extreme events (red dots) are based on the E-index, the western central Pacific (WCP, orange dots), EP (blue dots) and eastern central Pacific (ECP, gray dots) are based on the pioneering work of ref.[10]. **(e)** The extreme, EP and CP events are based on the Niño method.

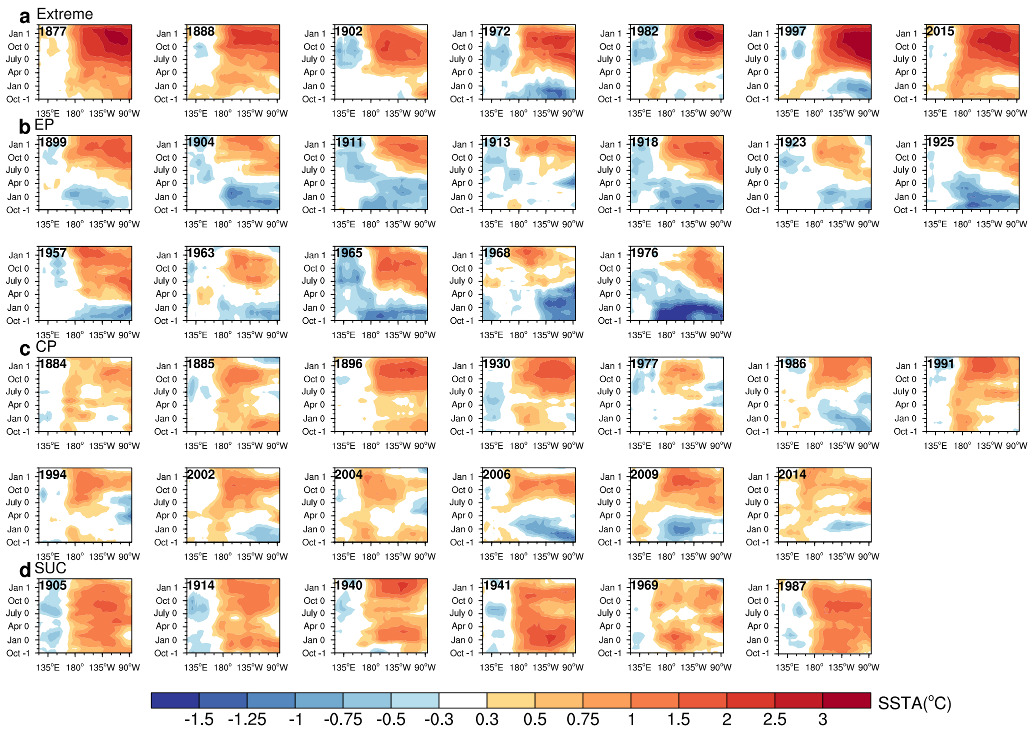

Supplementary Fig. 3 The sea surface temperature anomalies (SSTA) evolution patterns for each individual El Niño event within each cluster of El Niño events. a extreme (7 events); **b** Eastern Pacific (EP, 12 events), **c** Central Pacific (CP, 13 events), and **d** successive El Niño (6 events). The merged HadISST, ERSST5, and Kaplan datasets from 1871 to 2017 were used (after removing small linear trends).

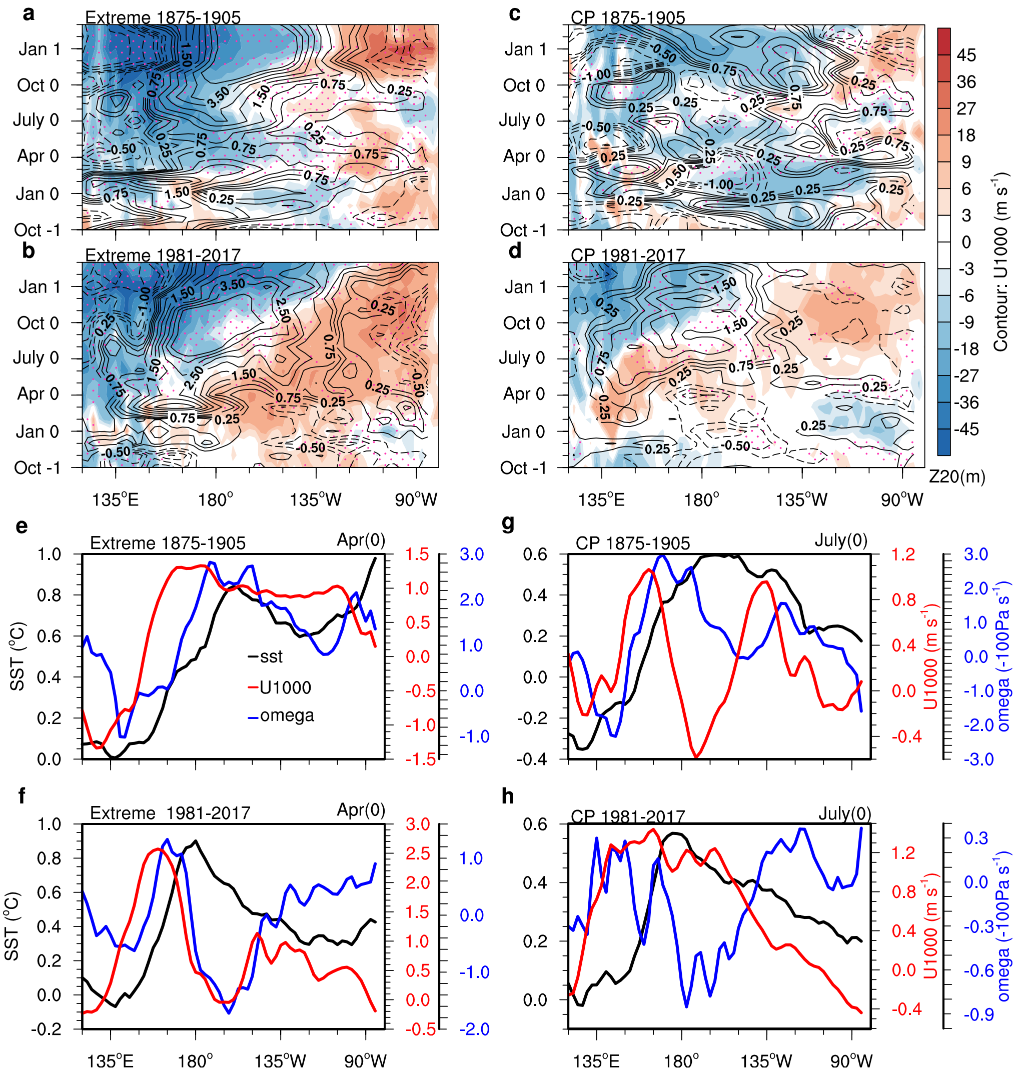

Supplementary Fig. 4 Comparison of surface zonal wind, thermocline, sea surface temperature, and convective anomaly associated with 2 types of El Niño events from 1875 to 1905 and 1981 to 2017. **a-b** Evolution of equatorial Pacific averaged (5°S-5°N) thermocline anomalies (shading units: °C) for extreme El Niño events for the period of 1875-1905 (**a**) and 1981-2017 (**b**). **c-d** Same as **a-b** but for Central Pacific (CP) El Niño events. Contours (units: m s^{-1}) denote the 1,000 hPa zonal wind anomaly (U1000), and color shading (in units of meters) denotes the thermocline depth anomaly (Z20). The stippling denotes the regions where the signal (group mean) is larger than noise (the SD of each member from the group mean) for zonal winds. **e-f** Composite of equatorial zonal SST (black line, units: °C), 1000 hPa zonal wind (red line, units: m s^{-1}), and convective (blue line, 500-hPa vertical motion, units: -100Pa s^{-1}) anomalies of extreme El Niño events at their onset time (Apial (0)) for the periods of 1875-1905 (**e**) and 1981-2017 (**f**). **g-h** Same as **e-f** but for CP El Niño events at their onset time (July (0)). The anomalies of each variable are calculated referenced to the climatology of the full period and linearly detrended. The merged HadISST, ERSST5, and Kaplan data from 1871 to 2017 were used (after removing small linear trends). For the zonal wind and omega, the merged National Centers for Environmental Prediction (NCEP) data were used.

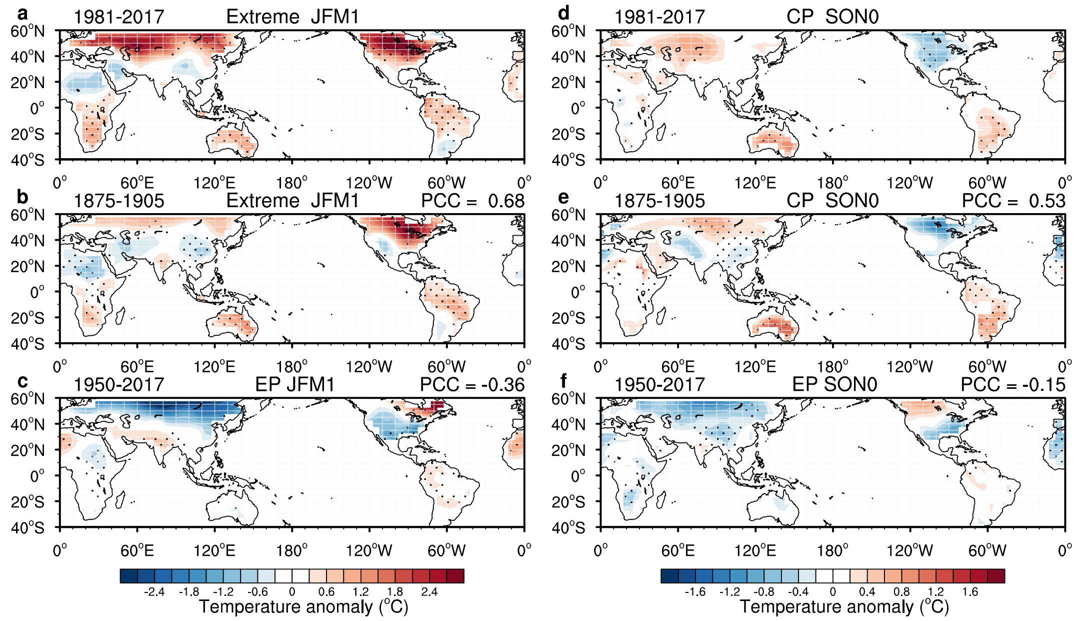

Supplementary Fig. 5 Global air temperature changes associated with El Niño diversity. **a-b** Composite of developing year winter to spring (January to March) (JFM1) air temperature anomalies (shading, °C) induced by extreme El Niño events for the periods of 1981-2017 **(a)** and 1875-1905 **(b)**. The spatial pattern correlation coefficient (PCC) between a and b is 0.68. **c** Similar to **a** but for EP El Niño events during the period of 1950-2017. We compare the spatial pattern correlation between **a** and **c** to show that the impacts of extreme El Niño events are different from those of EP El Niño events (PCC = -0.36). **d-e** Same as **a-b** but for the El Niño developing year autumn (SON0) of CP El Niño events. **f** Similar to **c** but for the El Niño developing year autumn (SON0). The spatial pattern correlation coefficient between **d** and **e** (**f**) is 0.53 (-0.15). The stippling denotes the regions where the signal (group mean) is larger than the noise (one standard deviation from the group mean of each member). Temperature anomalies are derived from HadCRUTV5 relative to the 1961-1990 means.

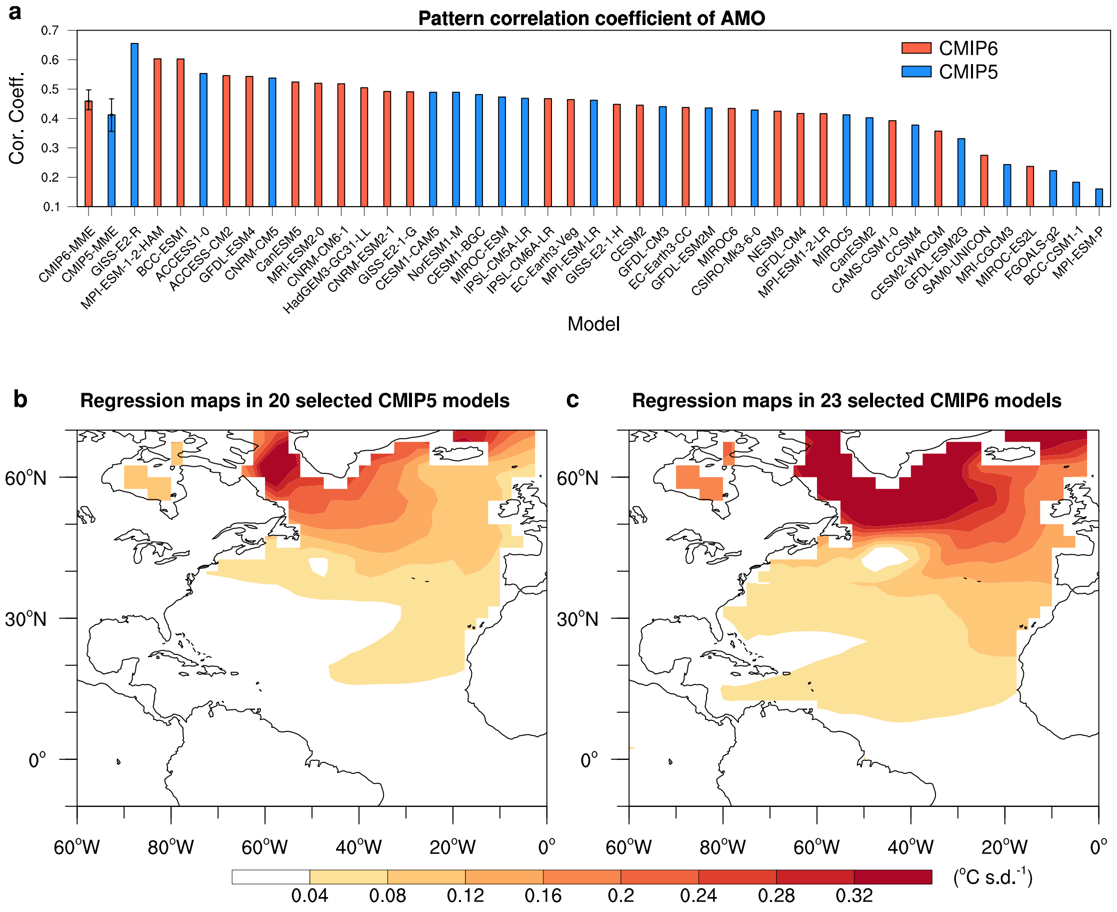

Supplementary Fig. 6 Assessment of Atlantic Multidecadal Oscillation (AMO) simulation in CMIP5 and CMIP6 models. a AMO metrics for picontrol simulations of CMIP5 (blue bars) and CMIP6 (orange bars) models, including pattern correlation coefficient (Cor. Coeff.) of each modelled AMO sea surface temperature (SST) variability pattern (0°S-70°N, 80°W-0°W). The error bar in the multimodel mean represents the 95% confidence level determined by a bootstrap test. **b-c** Spatial pattern of the Atlantic response by regression of SST (°C s.d.$^{-1}$; shaded) anomalies onto the normalized AMO index based on the multimodel average of the 20 selected CMIP5 models **(b)** and 23 selected CMIP6 models **(c)**.

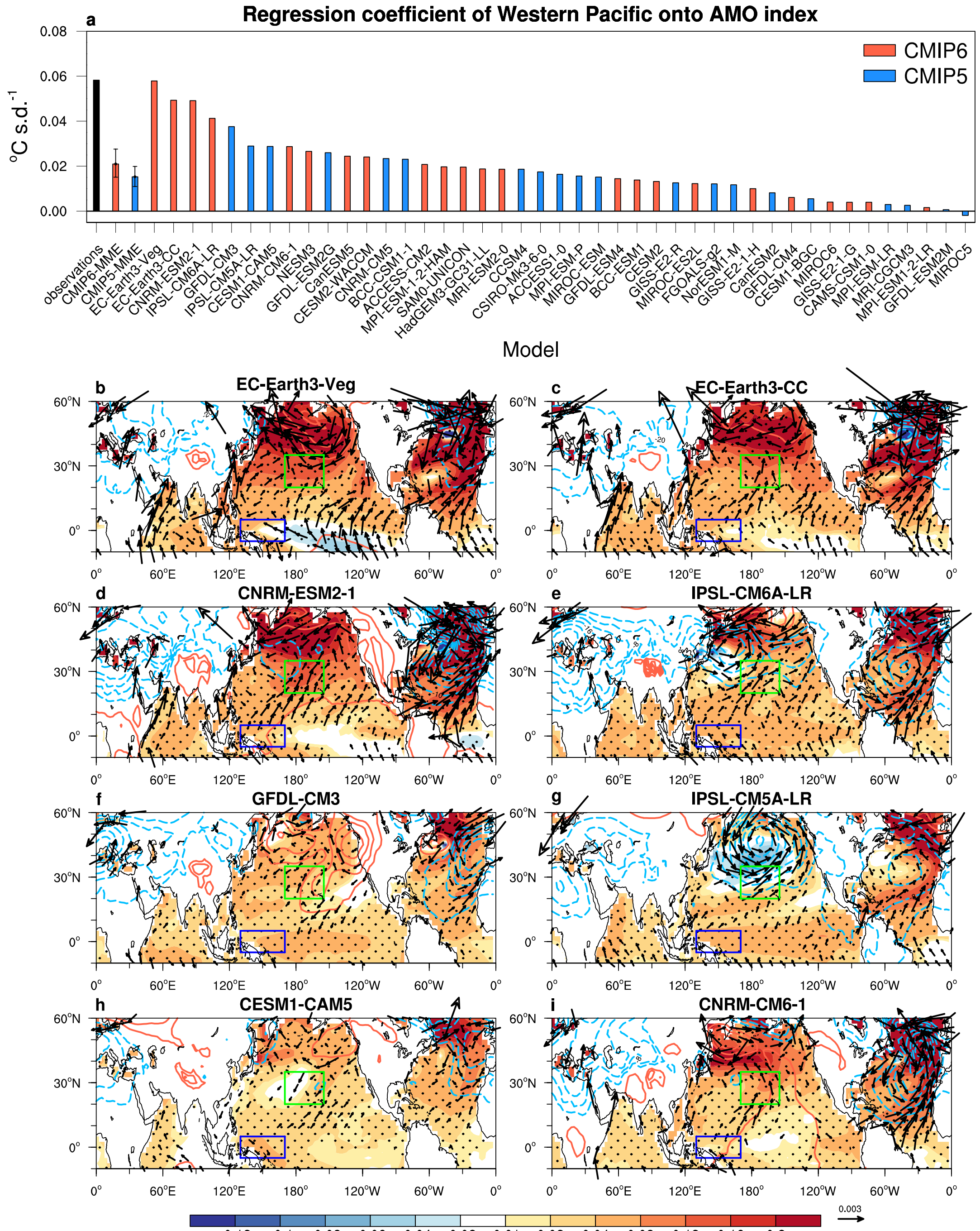

Supplementary Fig. 7 Modelled Pacific response to the Atlantic Multidecadal Oscillation (AMO). a Annual mean western Pacific (WP) sea surface temperature (SST) response (°C s.d.$^{-1}$) to annual mean AMO index over the 1871–2017 period in observations (black bar) and over the last 300 years in CMIP6 (orange bars) and CMIP5 (blue bars) models, measured by area-averaged regression coefficients of the grid-point WP SST anomalies (5°S-25°N, 130°E-170°E) onto the normalized AMO index. The error bar in the multimodel mean represents the 95% confidence level determined by a bootstrap test. **b-i** Spatial pattern of the Pacific response by regression of decadal Pacific SST (°C s.d.$^{-1}$; shaded), surface wind stress vectors (N m^{-2}; vectors), and sea level pressure (Pa; contours) anomalies onto the normalized AMO index based on 5

CMIP6 and 3 CMIP5 models that simulate WP SST responses comparable to the AMO with those in the observations. All data are applied to a 21-year running mean filter.

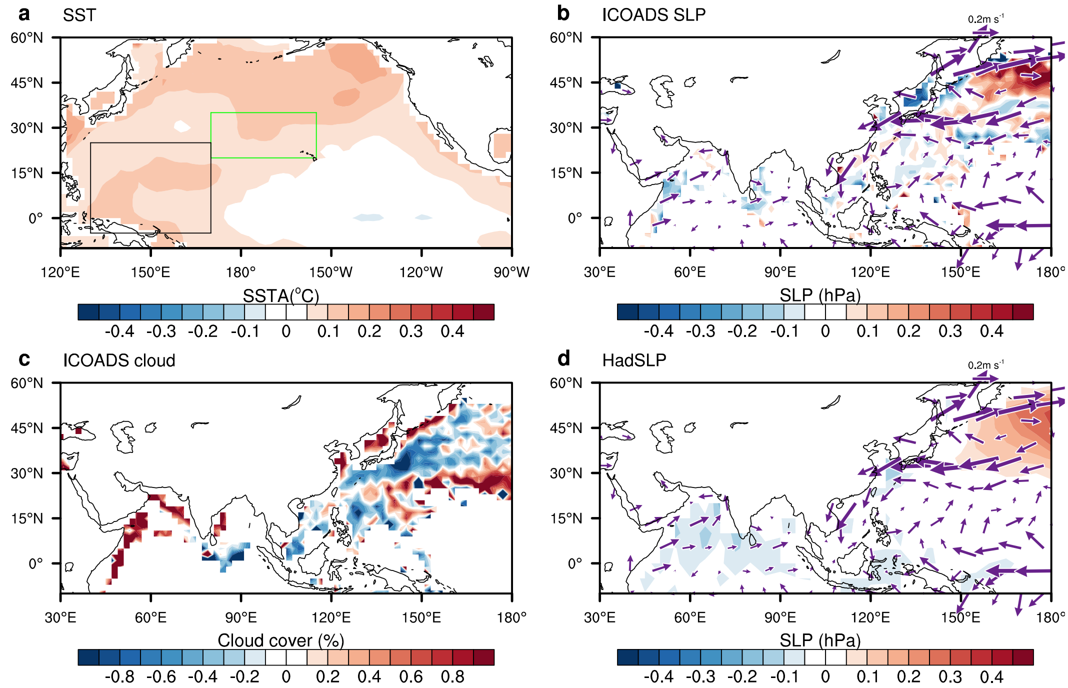

Supplementary Fig. 8 Pacific response to the Atlantic Multidecadal Oscillation (AMO) in Observation. a Regressions of the Pacific sea surface temperature anomaly (SSTA) (units: °C) on the normalized annual mean AMO index for 1871-2017 at decadal time scales. The green box represents the subtropical North Pacific (SNP) region (20°N–35°N, 170°E–155°W) and the black box represents Western Tropical Pacific (WTP) region (5°S-25°N, 130°E-170°E). The merged sea surface temperature (SST) data was used. **b** Same as **a** but for ICOADS sea level pressure (SLP, shading units: hPa) and for 1,000 hPa wind (vector, units: m s^{-1}). **c** Same as **a** but for cloud cover (units: %). **d**, the same as **b** but the SLP data derived from the HadSLP dataset. All data are applied to a 21-year running mean filter. The linear trends are removed in all variables. For the wind, the merged National Centers for Environmental Prediction (NCEP) dataset was used.

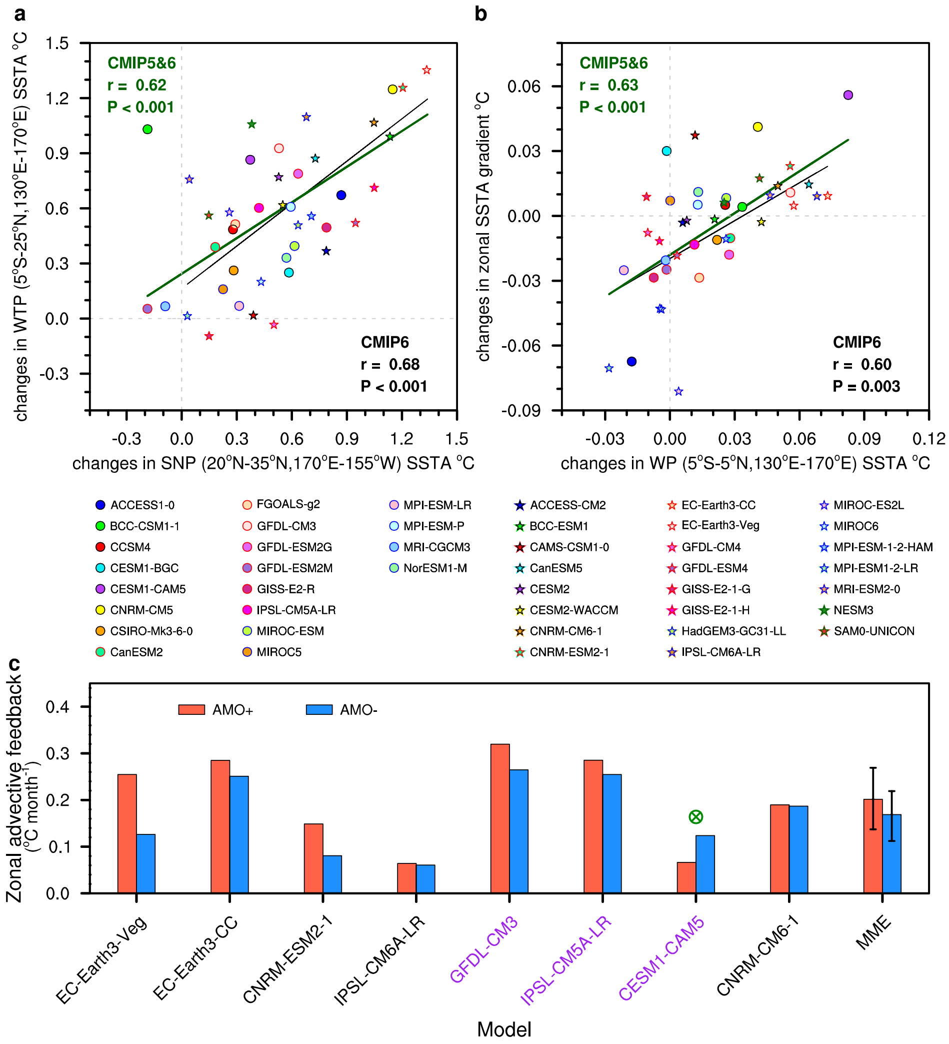

Supplementary Fig. 9 Mechanism for Atlantic Multidecadal Oscillation (AMO) forcing on El Niño diversity. a Inter-model relationship between the change in 21-year running mean subtropical North Pacific (20°S-35°N, 170°E-155°W, SNP) sea surface temperature anomalies (SSTA) (x-axis, units: °C) and the change in 21-year running mean Western Tropical Pacific (5°S-25°N, 130°E-170°E, WTP) SSTA (y-axis, units: °C) for the AMO positive state (AMO-positive state) minus AMO-positive state. **b** Same as **a** but for the inter-model relationship between the change in 21-year running mean western Pacific (WP, 5°S-5°N, 130°E-170°E) SSTA and 21-year running mean zonal sea surface temperature (SST) gradient [WP SST (5°S-5°N, 155°E-175°W) minus central Pacific SST (5°S-5°N, 115°W-145°W)]. **c** Zonal advective feedback term of El Niño events during the development phase (April, May, June (AMJ)) averaged over the central-eastern Pacific (5°S-5°N, 180°-80°W) for the AMO-positive state (AMO+, red bars) and AMO-negative state (AMO-, blue bars). The error bars in the multimodel mean represent the 95% confidence level determined by a bootstrap test. A total of 7 out of the 8 selected models (87.5%) simulate increased zonal advective feedback for El Niño events during the AMO-positive state compared to the AMO-negative state. Models that simulate a decrease are indicated by green circles. Models from CMIP5 are indicated in purple.

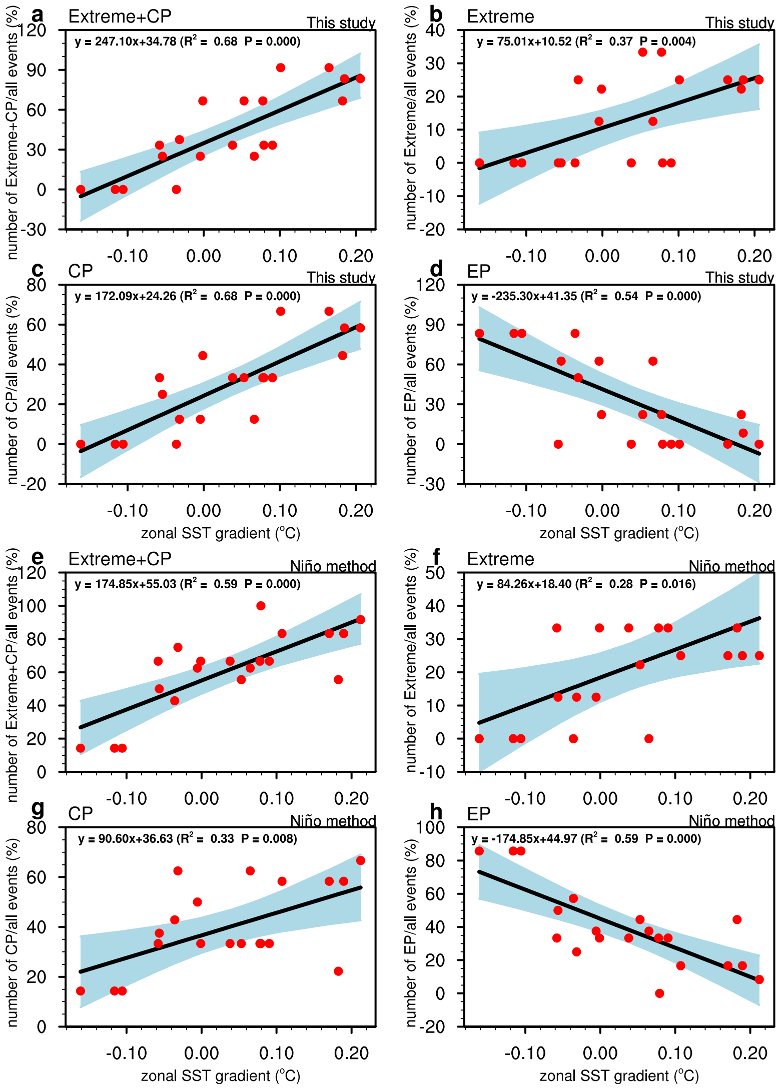

Supplementary Fig. 10 Simple linear regression model. a Relationship between the mean-state zonal sea surface temperature anomaly (SSTA) gradient during the 5 different periods (1875-1905, 1906-1929, 1930-1949, 1950-1980, 1981-2017) marked in Fig. 2a and its corresponding ratio of the sum of extreme and Central Pacific (CP) (a) to total El Niño events. **b-d** Similar to **(a)** but for the extreme **(b)**, the CP **(c)**, and the Eastern Pacific (EP) **(d)**. **e-h** Same as a-d but the extreme, CP and EP events are identified based on the Niño method. We used the HadISST, ERSSTV5, and Kaplan datasets shown in Supplementary Fig. 2a-c and the HadISST, ERSSTV5, and Kaplan merged sea surface temperature (SST) datasets shown in Fig. 2a. The mean-state zonal SSTA gradient is calculated as the 31-year running mean, annual-mean zonal equatorial SST anomalies (°C, relative to 1901-2010 mean) gradient (SSTA (5°S–5°N, 135°E–165°E) minus SSTA (5°S–5°N, 175°W–145°W)) in five periods. The black line represents the fit line, and the blue band shows the 95% confidence interval.

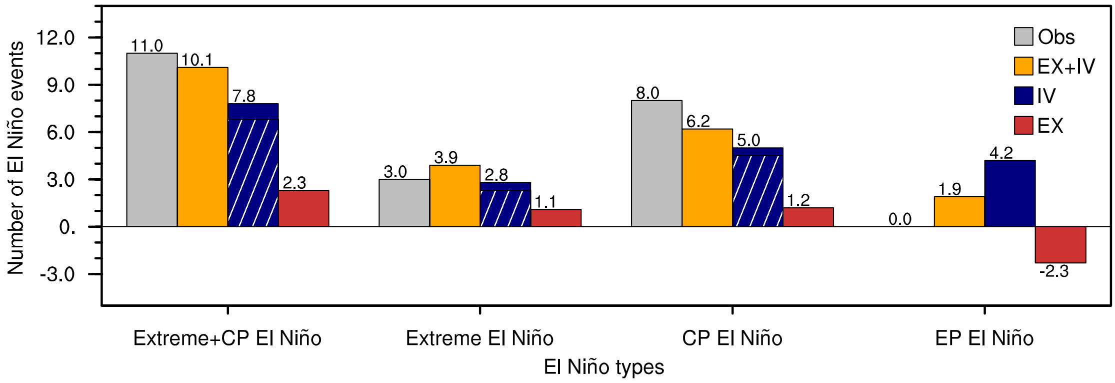

Supplementary Fig.11 Same as Figure 4b but the extreme El Niño, Central Pacific (CP) El Niño, and Eastern Pacific (EP) El Niño events are categorized according to the Niño method. Histograms for the estimated numbers of different types of El Niño events in 1981-2017 based on a linear regression model (see Methods and Supplementary Fig. 10e-f). The orange, blue, and red bars denote the estimated externally forced signal (EX) plus the internal variability signal (IV), IV, and EX-induced numbers of different types of El Niño events, respectively. Slant hatching denotes the Atlantic Multidecadal Oscillation (AMO) induced numbers of different types of El Niño events.

Supplementary References

1. Rayner NA, Parker DE, Horton EB, Folland CK, Alexander LV, Rowell DP*, et al.* Global analyses of sea surface temperature, sea ice, and night marine air temperature since the late nineteenth century. *J Geophys Res Atmos* 2003, **108**(D14).

2. Huang B, Thorne PW, Banzon VF, Boyer T, Chepurin G, Lawrimore JH*, et al.* Extended Reconstructed Sea Surface Temperature, Version 5 (ERSSTv5): Upgrades, Validations, and Intercomparisons. *J Climate* 2017, **30**(20)**:** 8179-8205.

3. Kaplan A, Cane MA, Kushnir Y, Clement AC, Blumenthal MB, Rajagopalan B. Analyses of global sea surface temperature 1856–1991. *Journal of Geophysical Research: Oceans* 1998, **103**(C9)**:** 18567-18589.

4. Ham Y-G, Kug J-S. How well do current climate models simulate two types of El Nino? Climate Dyn 39, 383-398 (2012).

5. Kug J-S, Jin F-F, An S-I. Two Types of El Niño Events: Cold Tongue El Niño and Warm Pool El Niño. J Climate 22, 1499-1515 (2009).

6. Feng JX, Wu ZH, Zou XL. Sea Surface Temperature Anomalies off Baja California: A Possible Precursor of ENSO. *J Atmos Sci* 2014, **71**(5)**:** 1529-1537.

7. Cai W, Wang G, Dewitte B, Wu L, Santoso A, Takahashi K*, et al.* Increased variability of eastern Pacific El Nino under greenhouse warming. *Nature* 2018, **564**(7735)**:** 201-206.

8. Wang G, Cai W, Santoso A. Stronger Increase in the Frequency of Extreme Convective than Extreme Warm El Niño Events under Greenhouse Warming. *J Climate* 2020, **33**(2)**:** 675-690.

9. Takahashi K, Dewitte B. Strong and moderate nonlinear El Niño regimes. *Climate Dyn* 2015, **46**(5-6)**:** 1627-1645.

10. Dommenget D, Bayr T, Frauen C. Analysis of the non-linearity in the pattern and time evolution of El Niño southern oscillation. *Climate Dyn* 2012, **40**(11-12)**:** 2825-2847.
